# Experience and perpetration of intimate partner violence and abuse by gender of respondent and their current partner before and during COVID-19 restrictions in 2020: a cross-sectional study in 13 countries

**DOI:** 10.1186/s12889-022-14635-2

**Published:** 2023-02-13

**Authors:** Gail Gilchrist, Laura C. Potts, Dean J. Connolly, Adam Winstock, Monica J. Barratt, Jason Ferris, Elizabeth Gilchrist, Emma Davies

**Affiliations:** 1 National Addiction Centre, Institute of Psychiatry, Psychology and Neuroscience, King’s College London, London, UK; 2 Department of Biostatistics and Health Informatics, Institute of Psychiatry, Psychology and Neuroscience, King’s College London, London, UK; 3 Barts Health NHS Trust, Whipps Cross University Hospital, London, UK; 4 Institute of Epidemiology and Health Care, University College London, London, UK; 5 Global Drug Survey, London, UK; 6 Social and Global Studies Centre and Digital Ethnography Research Centre, RMIT University, Melbourne, Australia; 7 National Drug and Alcohol Research Centre, UNSW Sydney, Randwick, Australia; 8 Centre for Health Services Research, Faculty of Medicine, The University of Queensland, Brisbane, Australia; 9 School of Health in Social Sciences, University of Edinburgh, Edinburgh, UK; 10 Centre for Psychological Research, Faculty of Health and Life Sciences, Oxford Brookes University, Oxford, UK

**Keywords:** Intimate partner violence and abuse, COVID-19, Gender and sexual minorities, Non-binary, LGBTQI+, Transgender

## Abstract

**Background:**

Intimate partner violence and abuse (IPVA) includes controlling behaviours, psychological, physical, sexual and financial abuse. Globally, surveys and emergency services have recorded an increase in IPVA since restrictions were imposed to limit COVID-19 transmission. Most studies have only included heterosexual women.

**Methods:**

Data from the Global Drug Survey (an annual, anonymous, online survey collecting data on drug use) Special Edition were analysed to explore the impact of COVID-19 on people’s lives, including their intimate relationships. Five relationship groupings were created using respondents’ lived gender identity: women partnered with men (46.9%), women partnered with women (2.1%), men partnered with men (2.9%), men partnered with women (47.2%), and partnerships where one or both partners were non-binary (1%). Self-reported experience and perpetration of IPVA in the past 30 days before (February) and during COVID-19 restrictions (May or June) in 2020 (*N* = 35,854) was described and compared for different relationship groupings using Fishers Exact Tests. Changes in IPVA during restrictions were assessed using multivariable logistic regression.

**Results:**

During restrictions, 17.8 and 16.6% of respondents had experienced or perpetrated IPVA respectively; 38.2% of survivors and 37.6% of perpetrators reported this had increased during restrictions. Greater proportions of non-binary respondents or respondents with a non-binary partner reported experiencing or perpetrating IPVA (*p* < .001) than other relationship groupings. 22.0% of respondents who were non-binary or had a non-binary partner, 19.5% of men partnered with men, 18.9% of men partnered with women, 17.1% of women partnered with women and 16.6% of women partnered with men reported experiencing IPVA. Respondents with higher psychological distress, poor coping with pandemic-related changes, relationship tension and changes (increases or increases and decreases) in alcohol consumption reported increased experience of IPVA during restrictions.

**Conclusions:**

This study confirmed that IPVA can occur in all intimate relationships, regardless of gender of the perpetrator or survivor. Non-binary respondents or respondents with non-binary partners reported the highest use and experience of IPVA. Most IPVA victim support services have been designed for heterosexual, cisgender women. IPVA support services and perpetrator programmes must be tailored to support all perpetrators and survivors during the pandemic and beyond, regardless of their sexual or gender identity.

**Supplementary Information:**

The online version contains supplementary material available at 10.1186/s12889-022-14635-2.

## Introduction

Intimate partner violence and abuse (IPVA) refers to any behavior perpetrated by a current or ex-partner causing physical, sexual or psychological harm [1]. When restrictions were imposed to limit COVID-19 transmission, an increase in IPVA was recorded in surveys and by emergency services globally [2–17]. Studies of cohabiting or partnered women reported that 13–65% reported experiencing IPVA during COVID-19 [3–7, 9, 10]. Pre-pandemic reviews have reported pooled prevalences of ever experiencing physical or sexual IPVA of 27% (physical and/or sexual IPVA) among heterosexual women [18], 19% (physical IPVA only) among heterosexual men [19], 17 and 9% respectively among men who have sex with men (MSM) [20] and 38 and 25% respectively among transgender people [21]. Stay at home measures and lockdowns provided perpetrators the opportunity to isolate and control their partners, who had reduced access to social support, all of which may have contributed to an escalation in IPVA [15, 22]. In addition, restrictions may have impacted known risk factors for IPVA such as substance use and mental health problems, which may have increased the risk of IPVA perpetration [23–26].

Higher rates of IPVA were reported in studies conducted during the pandemic in some low-middle income countries (45%, Bangladesh [6]; 65%, Iran [5]) but not others (15%, Tunisia [10]; 18%, India [4]). Lower rates were generally, but not always (59%, Argentina [7]),reported in high income countries (13%, Australia [2]; 16%, US [3]; 8%, Germany [9]), with similar rates reported by cohabiting or partnered MSM (13–15%, US [15, 16]). Most of the abovementioned studies, conducted during the pandemic, included heterosexual women only [2, 4–6, 9, 10], two explored the experiences of men who have sex with men (MSM) [15, 16] and two explored the experiences of both men and women [3, 17]. Only three studies described how ‘gender’ was determined [3, 15, 17]. One used ‘sex at birth’ and gave no justification for choosing this over lived gender [3], one included cisgender (cis) men [15] and one recorded both sex assigned at birth and gender identity [17]. In one study, where half the sample identified as a sexual or gender minority (SGM), comparisons between cis heterosexuals and SGM respondents were not reported for IPVA [17].

Of the two studies that included both men and women, only one reported findings separately [3]. This US study found 23% of men and 15% of women had experienced IPVA during COVID-19 restrictions [3]. Respondents in several studies reported an increase or worsening in experiencing IPVA during COVID-19 restrictions [2–5, 10, 15–17]. COVID-19 related stress including negative economic changes, increased caring responsibilities and quarantine were associated with (new) exposure to IPVA [3–5, 7–9, 17]. The risk of experiencing IPVA during lockdown increased for women who had previously experienced IPVA [10]. Among MSM, lower education, increased substance use, higher levels of anxiety and additional sexual partners (among those with non-monogamous sexual agreements) were associated with experiencing (new) IPVA during lockdown [16, 17].

We are not aware of any studies conducted during COVID-19 restrictions among other SGM. This survey was the first to explore the influence of gender in more detail by comparing the use and experience of IPVA by gender of respondent and their current partner before and during COVID-19 restrictions in 2020, required to inform the response to IPVA.

## Methods

### Aims

The aims of this manuscript are to 1) describe and compare the self-reported experience and perpetration of abusive behaviors, and the overlap in these behaviors in the past 30 days (during COVID-19 restrictions, May–June 2020) by gender identity of respondent and their partner; 2) compare and describe changes in abusive behaviors during a month of COVID-19 restrictions compared to February 2020 by gender identity of respondent and their partner; and 3) explore factors associated with an increase in abusive behaviors during COVID-19 restrictions.

### Survey

Since 2012, the anonymous, online international, annual Global Drug Survey (GDS).

includes a core set of questions on demographics, alcohol use, illicit and licit drug use and mental health, providing continuity and comparability across surveys. In addition, each GDS includes specialist topics [27]. GDS recruitment partners include The Guardian and Vice (UK), Zeit Online (Germany) and Stuff.nz (New Zealand). Cross-promotion through social network channels was also encouraged. As a result, the GDS sample is not representative, with respondents being younger and more experienced with illicit drugs. A lack of ethnic diversity in the sample has also been described. Despite this, comparisons with general population surveys in Australia, Switzerland and the US found that GDS respondents had similar demographic characteristics to those who use cannabis and alcohol in their country [27]. The GDS Special Edition on COVID-19 was available in 10 languages during 3 May–21 June 2020 to explore the pandemic’s impact on substance use, mental health and relationships. No information is available on how long the survey took to complete from users. However, respondents were advised at the beginning of the survey “Participation in the survey will take about 15 minutes if you only drink alcohol, and another 10 minutes if you use other drugs”. The true length of time to complete was determined by the number of different drugs consumed.

### Ethics and safety

University College London granted ethical approval (11,671/001).

### Sample

The original data is a non-probability sample of 59,969 responses from 171 countries. Data were restricted to countries with 500 respondents or more (allowing sufficient in-country heterogeneity across the modelled variables); resulting in a sample of 56,927 from 13 countries (Australia, Austria, Brazil, Denmark, France, Germany, Greece, Ireland, Netherlands, New Zealand, Switzerland, UK, US). The sample was further restricted to respondents with (at least) one partner (*N* = 35,984). Analysis exploring relationship group differences included only those reporting their current partner’s gender (N = 35,854), resulting in some countries having less than 500 respondents (Table 1).

**Table 1:** Sample characteristics (*n* = 35,984)

Respondent		n (%)
Age (years) – mean (sd) N=35,984/ Missing=0		37.1 (13.0)
Gender identity N=35,984/ Missing=0	Male	18,078 (50.2)
	Female	17,658 (49.1)
	Non-binary	248 (0.7)
Gender (includes gender assigned at birth) N=35,600/ Missing=384	Cis man	17,848 (50.1)
	Cis woman	17,388 (48.8)
	Transgender man	58 (0.2)
	Transgender woman	58 (0.2)
	Non-binary	248 (0.7)
Sexual orientation N=35,913/ Missing = 71	Straight or heterosexual	31,207 (86.9)
	Lesbian, gay or homosexual	1410 (3.9)
	Bisexual	2248 (6.3)
	Queer	369 (1.0)
	Different orientation	151 (0.4)
	Don’t know/prefer not to say	528 (1.5)
Ethnicity *N* = 32,368/ Missing = 3616	White	30,299 (93.6)
	Black / African American	174 (0.5)
	Asian	183 (0.6)
	Hispanic/Latino	349 (1.1)
	Mixed	1025 (3.2)
	Other	338 (1.0)
Country of residence N = 35,984// Missing = 0	Australia	969 (2.7)
	Austria	678 (1.9)
	Brazil	1991 (5.5)
	Denmark	267 (0.7)
	France	3604 (10.0)
	Germany	17,120 (47.6)
	Greece	223 (0.6)
	Ireland	3224 (9.0)
	Netherlands	1680 (4.7)
	New Zealand	2114 (5.9)
	Switzerland	2335 (6.5)
	United Kingdom	1356 (3.8)
	United States	423 (1.2)
Currently live with children N = 32,474/ Missing = 3510	No	20,234 (62.3)
	Yes	12,240 (37.7)
Area of residence *N* = 33,002/ Missing = 2982	City/urban area	23,572 (71.4)
	Regional area	7784 (23.6)
	Remote/rural area	1646 (5.0)
Are you currently living in your regular place of residence? N = 32,973/ Missing = 3011	Yes	31,075 (94.2)
	No, I relocated voluntarily due to the COVID-19 pandemic/restrictions	1184 (3.6)
	No, I am stranded by the COVID-19 movement restrictions	199 (0.6)
	No, but this situation is unrelated to COVID-19	515 (1.6)
Are you currently in paid employment? N = 35,573/ Missing = 411	No	7605 (21.4)
	Yes, full-time	20,687 (58.2)
	Yes, part-time / casual	7281 (20.5)
Has the amount of money you have left after expenses changed compared with before COVID-19? N = 35,614/ Missing = 370	No change	17,440 (49.0)
	I/ We have more now	8065 (22.6)
	I/ We have less now	10,109 (28.4)
In the past month, how difficult has it been for you to pay for the very basics like food, housing, medical care, and heating? N = 35,766/ Missing = 218	Not very difficult	30,429 (85.1)
	Somewhat difficult	3636 (10.2)
	Difficult	1227 (3.4)
	Very difficult	474 (1.3)
How have you been coping with changes related to the COVID-19 pandemic? N = 35,882/ Missing = 102	I’m coping really well	18,278 (50.9)
	I’m coping with some things but not others	16,739 (46.7)
	I’m not coping well at all	865 (2.4)
Kessler 6 Distress Scale total score [median (LQ - UQ)] *N* = 27,919/ Missing = 8065	5 (3–9)	6.4 (4.7)
Severe Psychological Distress N = 27,919/ Missing = 8065	≥13	3355 (12.0)
	< 13	24,564 (88.0)
Alcohol change (composite) *N* = 34,819/ Missing = 1165	Only decreased	8992 (25.8)
	Only increased	14,078 (40.4)
	Increased and decreased	2607 (7.5)
	No change	9142 (26.3)
Tension in relationship before COVID-19 restrictions N = 27,305/ Missing = 8679	No tension	16,061 (58.8)
	Some tension	9877 (36.2)
	A lot of tension	1367 (5.0)
Tension in relationship during COVID-19 restrictions N = 27,962/ Missing = 8022	No tension	14,332 (51.3)
	Some tension	11,437 (40.9)
	A lot of tension	2193 (7.8)
**Partner**		**n (%)**
Gender identity N = 35,854/ Missing = 130	Male	17,947 (50.1)
	Female	17,759 (49.5)
	Non-binary	148 (0.4)
Dyad gender grouping N = 35,854/ Missing = 130	Women partnered with women	747 (2.1)
	Women partnered with men	16,805 (46.9)
	Men partnered with women	16,915 (47.2)
	Men partnered with men	1042 (2.9)
	Non-binary respondents or respondents with a non-binary partner	345 (1.0)
Dyad gender grouping (includes gender assigned at birth) N = 35,419/ Missing = 565	Cis man-cis woman	16,615 (46.9)
	Cis man-cis man	956 (2.7)
	Cis woman-cis man	16,512 (46.6)
	Cis woman-cis woman	671 (1.9)
	Cis man-transgender/non-binary person	180 (0.5)
	Cis woman - transgender/non-binary person	125 (0.4)
	Transgender/non-binary person - cis man	147 (0.4)
	Transgender/non-binary person - cis woman	138 (0.4)
	Transgender/non-binary person – Transgender/non-binary person	75 (0.2)

### Measures

Additional file 1 Supplementary Table 1 describes the questions and response options for all survey questions reported in this analysis.

#### Socio-demographics

Data on age, gender, ethnicity, area of residence, living circumstances, employment and finances were collected.

#### Gender

Respondents were asked ‘What is your gender?’ (response options ‘male’, ‘female’, non-binary’ or ‘different identity’) followed by ‘What gender were you assigned at birth?’ (response options ‘male’ or ‘female’). They were also asked to provide the gender and gender assigned at birth for their current partner. If the respondents’ current lived gender was ‘male’ or ‘female’ and differed from the gender they were assigned at birth, they were defined as transgender. For example, if a respondent identified as male but was assigned a female gender at birth, they were defined as a transgender women for analysis. Respondents selecting ‘non-binary’ or ‘different identity’ were combined into the category, ‘non-binary person’. A full explanation of this two stage process is described elsewhere [28]. Respondents and their partners were assigned to one of five gender groups (i.e., cis man, cis woman, transgender man, transgender woman, non-binary person), resulting in nine unique relationship groups (e.g. cis men partnered with cis women, cis men partnered with transgender women, non-binary people partnered with cis women, etc.). Cell counts were very small for some relationship groupings, precluding meaningful analysis of IPVA in pairings where one partner was a gender minority.

We used respondents’ lived gender identity to create five relationship groups for statistical comparisons: women partnered with men, women partnered with women, men partnered with men, men partnered with women, and partnerships where one or both partners were non-binary. While acknowledging that some people identify strictly as transgender men/women rather than men/women (and may have been miscategorised by selecting ‘different identity’), we felt that respecting and prioritising as many respondents’ lived gender identity was most important [29]. The alternative, based on the available cell counts, was to group all transgender and/or non-binary people together. However, we believe this was invalid because of the heterogeneity in gender identity, presentation, gender minority stress and IPVA risk factors among transgender and/or non-binary people. We felt that differences between binary transgender and cis gender identities are often no more significant than the diversity that exists across the spectrum of individual cis gender identities and so grouped binary transgender and cis respondents together (i.e., cis women with transgender women).

While non-binary respondents are similarly diverse in the aforementioned (and other) characteristics, there was insufficient data to further subcategorise. Working on the premise that the majority of those identifying with ‘non-binary’ or ‘different identity’ reject assignment to binary gender categories, we agreed that this shared identity and potential stressor was a significant enough commonality for grouping.

#### Intimate relationships

Intimate relationships were defined as having a husband/wife, partner or boyfriend/girlfriend for longer than one month. Tension in intimate relationships (no tension; some tension; a lot of tension) in February 2020, before the COVID-19 restrictions; and in the past 30 days (during May–June 2020) was measured using the question ‘In general, how would you describe your relationship’ with your partner/boyfriend/girlfriend in the last 30 days, from the Woman Abuse Screening Tool-Short [30]. The 15-item Composite Abuse Scale Revised–Short Form (CASR-SF) assesses lifetime and past 12 months experience of IPVA among women [31]. Internal consistency of the CASR-SF is 0.942 [21]. Eight CASR-SF items were selected (seven remained unchanged and one ‘Harassed me by phone, text, email or using social media’ was changed to ‘checked up on me by checking my phone, text, email or social media without my consent’) to assess experience and adapted to measure perpetration of *financial abuse* (kept me from having access to a job, money or financial resources); *emotional abuse* (told me were crazy, stupid or not good enough); *coercive control* (kept me from seeing or talking to family or friends); *threatening behavior* (threatened to harm or kill partner or someone close to me); *technology-facilitated abuse* (checked up on me by checking phone, text, email or social media without consent); *physical abuse* (shook, pushed, grabbed or threw me; and/or hit me with a fist or object, kicked or bit me); and *sexual abuse* (forced or tried to force me to have sex). For example, the CASR-SF includes the item ‘My partner(s) shook, pushed, grabbed or threw me’ which we adapted to **‘**my partner/boyfriend/girlfriend shook, pushed, grabbed or threw me’ for experience of IPVA and ‘I shook, pushed, grabbed or threw my partner/boyfriend/girlfriend’ for perpetration of IVPA. The adapted CASR-SF assessed frequency (not in the past 30 days; once; a few times (2–3); weekly/ almost weekly; daily/ almost daily) of experiencing and perpetrating IPVA in the past 30 days preceding survey completion. Binary variables were created to present whether any of the abuse types were present during the past 30 days (yes/no). For each of the eight abusive behaviors, respondents who had experienced these, were asked whether this behavior had increased, stayed the same or decreased compared to February, before the COVID-19 restrictions.

#### Alcohol

Three measures assessed changes in alcohol consumption compared to February, before COVID-19 restrictions, related to frequency of consumption in days, number of standard drinks consumed on a typical day, and frequency of consuming more than five drinks on a single occasion (measure of binge drinking). Response options were: increased a lot, increased a little, stayed the same, decreased a little, decreased a lot. A composite variable to capture overall changes in consumption was created to group respondents into those who had increased on all three measures, decreased on all three measures, stayed the same, or who had responded that they had increased in one or two measures, and decreased in the other, or decreased in one or two measures and increased in the other.

#### Mental health and coping

The six-item Kessler Psychological Distress Scale (K-6) assessed how often (from 0 (none of the time) to 4 (all of the time)) respondents felt nervous, hopeless, restless or fidgety, so sad that nothing could cheer them up, that everything was an effort, and worthless, in the past 30 days [32]. The K-6 was developed for use in population studies. Internal consistency of the K-6 is 0.89 [22]. Total scores ranged from 0 to 24, with higher scores indicating higher distress. Scores of 5–13 are indicative of moderate mental distress [33] and scores of at least 13 are indicative of severe psychological distress [34]. Respondents were asked ‘How have you been coping with changes related to the COVID-19 pandemic?’ with response options ‘I’m coping really well’, ‘I’m coping with some things but not others’, ‘I’m not coping well at all’.

### Statistical analysis

Complete case analyses were performed using Stata (version 16). Missing data were not imputed. A complete case approach was taken under the assumption that respondents with missing data were a random sample of the full dataset. We acknowledge this approach may have led to bias in estimates. Descriptive statistics for the sample characteristics, binary measures of the self-reported experience and perpetration of IPVA during COVID-19 restrictions, and changes in IPVA compared to February 2020 (pre-COVID restrictions) were reported. Continuous symmetric measures were described using mean and standard deviation (sd) for non-skewed data and using medians and interquartile ranges (IQRs) for skewed data. Categorical measures were described using both numbers and proportions. Bonferroni adjustments corrected for multiple testing [35].

A selection of a priori variables were tested for a relationship with any increase in experiencing IPVA (out of those who reported experiencing any IPVA type) and any increase in perpetrating IPVA (out of those who reported perpetration of any IPVA type) using logistic regression. These variables were: age, K-6 total score, relationship groups (women partnered with women, women partnered with men, men partnered with women, men partnered with men and non-binary respondents or respondents with a non-binary partner), ethnicity (White; Black/ African American; Asian; Hispanic/Latino; Mixed; Other), lives with children (Yes; No), area of residence (Urban; Regional; Rural), currently living in regular place of residence (Yes; No, I relocated voluntarily due to the COVID-19 pandemic/ restrictions; No, I am stranded by the COVID-19 movement restrictions; No, but this situation is unrelated to COVID-19), change in money left after expenses compared with before the COVID-19 restrictions (No change; More disposable income; Less disposable income), difficulty in paying for the very basics in the past month (Not very difficult; Somewhat difficult; Difficult; Very difficult), coping with changes related to the pandemic (Coping really well; Coping somewhat well; Not coping well), relationship tension in past 30 days (No tension; Some tension; A lot of tension) and change in alcohol consumption (Only decreased; No change; Only increased/Increased and decreased). Variables with *p* < 0.2 in a univariable logistic regression were entered into a multivariable model to ascertain the variables associated with increases and decreases in IPVA, respectively [36]. This cut-off was chosen to purposively select clinically important as well as statistically significant variables for inclusion in the logistic regression [26].

## Results

### Sample characteristics

Table 1 describes respondents’ socio-demographic characteristics. Based on self-reported gender identity and that of their partner, 47.2% were men partnered with women; 46.9% were women partnered with men; 2.9% were men partnered with men; 2.1% were women partnered with women; 1% were relationships where the respondent and/or their partner identified as non-binary (non-binary group) (*N* = 35,854).

The average age of respondents was 37.1 years, the majority were white (93.6%), did not live with children (62.3%), lived in city/urban areas (71.4%), had not been displaced as a result of COVID-19 (94.2%) and were in part- or full-time paid employment (78.7%). The majority reported no difficulties in paying for the very basics (e.g., food, housing, medical care, and heating; 85.1%) and either no change or an increase (71.6%) in the amount of money they had after expenses in the past month during COVID-19 restrictions compared with before the COVID-19 restrictions. Around half of the sample reported coping really well (50.9%) with changes related to the COVID-19 pandemic.

### Mental health and alcohol use

The K-6 median (IQR) score was 5 (3–9). 12 % met criteria for severe psychological distress. There were significant differences by relationship groupings for severe psychological distress and changes in alcohol consumption during COVID-19 restrictions (Table 2). Non-binary respondents or respondents with non-binary partners presented the highest proportion of severe psychological distress (34.4%; *p* < 0.001). Of all relationship groupings, men partnered with men reported the highest proportion of increases in alcohol consumption during restrictions (40.4%; p < 0.001).

**Table 2:** Alcohol change and Psychological Distress by gender of respondent and their current partner (n = 35,854)

		Women partnered with women (*n* = 747)	Women partnered with men (*n* = 16,805)	Men partnered with women (n = 16,915)	Men partnered with men (*n* = 1042)	Non-binary respondents or respondents with a non-binary partner (*n* = 345)	Sample (n = 35,854)	*P* value (Chi 2 test)
Composite change in alcohol use - n(%)	Only decreased	205/714 (28.7)	4109/16329 (25.2)	4261/16327 (26.1)	290/998 (29.1)	90/323 (27.9)	8992/34691 (25.8)	*p* < .001
	No change	165/714 (23.1)	4353/16329 (26.7)	4301/16327 (26.3)	212/998 (21.2)	76/323 (23.5)	9142/34691 (26.3)	
	Only increased	280/714 (39.2)	6679/16329 (40.9)	6515/16327 (39.9)	428/998 (42.9)	125/323 (38.7)	14,078/34691 (40.4)	
	Increased and decreased	64/714 (9.0)	1188/16329 (7.3)	1250/16327 (7.7)	68/998 (6.8)	32/323 (9.9)	2607/34691 (7.5)	
Kessler 6 Distress Scale n(%)	Non-case	476/590 (80.7)	11,320/13313 (85.0)	11,807/12845 (91.9)	682/789 (86.4)	181/276 (65.6)	24,564/27813 (88.0)	*p* < .001
	Severe psychological distress (≥13)	114/590 (19.3)	1993/13313 (15.0)	1038/12845 (8.1)	107/789 (13.6)	95/276 (34.4)	3355/27813 (12.0)	
Kessler 6 total score - median (LQ – UQ)		7 (4–11)	7 (3–10)	4 (2–8)	6 (3–10)	10 (6–14)	5 (3–9)	–

### Intimate relationships

An increased proportion of respondents reported some or a lot of tension in their relationships during the past 30 days during COVID-19 restrictions (48.7%) compared to the month of February before such restrictions (41.2%; Table 1).

Overall, 10.5% reported both experiencing and using abusive behavior; 7.4% had only experienced abuse from their partner and 6.1% had only used abusive behavior towards their partner during COVID-19 restrictions (Table 3). There was a significant difference in IPVA by relationship grouping during COVID-19 restrictions: 22.0% of non-binary respondents or respondents with non-binary partners, 19.5% of men partnered with men, 18.9% of men partnered with women, 17.1% of women partnered with women and 16.6% of women partnered with men had experienced IPVA (*p* < 0.001); and 23.1% of non-binary respondents or respondents with non-binary partners, 17.5% of men partnered with men, 17.4% of women partnered with men, 15.5% of men partnered with women and 15.4% of women partnered with women had used IPVA (p < 0.001).

**Table 3:** Experience and use of intimate partner violence and abuse (IPVA) by gender of respondent and their partner gender of respondent and their partner during the past 30 days of COVID-19 restrictions (n = 35,854)

Variable, n (%)		Women partnered with women (n = 747)	Women partnered with men (n = 16,805)	Men partnered with women (n = 16,915)	Men partnered with men (n = 1042)	Non-binary respondents or respondents with a non-binary partner (n = 345)	Sample (n = 35,854)	*P *value from Fishers Exact Test
Experienced financial control	Yes	8/512 (1.6)	82/11296 (0.7)	160/10445 (1.5)	5/680 (0.7)	5/224 (2.2)	260/23157 (1.1)	< 0.001
Experienced emotional abuse	Yes	60/514 (11.7)	1436/11352 (12.6)	1264/10468 (12.1)	86/677 (12.7)	34/223 (15.2)	2880/23234 (12.4)	0.447
Experienced coercive control	Yes	16/514 (3.1)	280/11284 (2.5)	431/10410 (4.1)	17/675 (2.5)	14/222 (6.3)	758/23105 (3.3)	< 0.001
Experienced threatening behaviour	Yes	1/509 (0.2)	63/11227 (0.6)	55/10388 (0.5)	7/674 (1.0)	2/221 (0.9)	128/23019 (0.6)	0.278
Experienced technology facilitated abuse	Yes	18/509 (3.5)	283/11290 (2.5)	505/10419 (4.8)	36/675 (5.3)	13/223 (5.8)	855/23116 (3.7)	< 0.001
Experienced physical abuse (shook/pushed/ grabbed)	Yes	18/510 (3.5)	256/11262 (2.3)	265/10404 (2.5)	15/674 (2.2)	9/225 (4.0)	563/23075 (2.4)	0.136
Experienced severe physical abuse (hit with fist or object)	Yes	6/511 (1.2)	63/11246 (0.6)	173/10390 (1.7)	12/673 (1.8)	6/224 (2.7)	260/23044 (1.1)	< 0.001
Experienced sexual abuse	Yes	5/511 (1.0)	195/11231 (1.7)	117/10379 (1.1)	13/673 (1.9)	4/222 (1.8)	334/23016 (1.4)	0.002
Experienced any IPVA	Yes	89/519 (17.1)	1900/11446 (16.6)	1995/10538 (18.9)	133/682 (19.5)	50/227 (22.0)	4167/23412 (17.8)	< 0.001
Perpetrated financial control	Yes	5/508 (1.0)	52/11033 (0.5)	62/10254 (0.6)	0/667 (0.0)	0/218 (0.0)	119/22690 (0.5)	0.059
Perpetrated emotional abuse	Yes	45/505 (8.9)	1171/11017 (10.6)	1064/10216 (10.4)	80/666 (12.0)	35/219 (16.0)	2395/22623 (10.6)	0.049
Perpetrated coercive control	Yes	9/507 (1.8)	182/10962 (1.7)	165/10182 (1.6)	7/664 (1.1)	8/215 (3.7)	371/22530 (1.6)	0.159
Perpetrated threatening behaviour	Yes	2/509 (0.4)	27/10961 (0.2)	30/10190 (0.3)	3/661 (0.5)	2/217 (0.9)	64/22538 (0.3)	0.185
Perpetrated technology facilitated abuse	Yes	27/508 (5.3)	712/10980 (6.5)	407/10181 (4.0)	38/664 (5.7)	15/216 (6.9)	1199/22549 (5.3)	< 0.001
Perpetrated physical abuse (shook/pushed/ grabbed)	Yes	16/506 (3.2)	182/10960 (1.7)	204/10178 (2.0)	10/665 (1.5)	7/217 (3.2)	419/22526 (1.9)	0.028
Perpetrated any severe physical abuse (hit with fist or object)	Yes	9/509 (1.8)	107/10948 (1.0)	68/10184 (0.7)	7/661 (1.1)	5/215 (2.3)	196/22517 (0.9)	0.002
Perpetrated sexual abuse	Yes	3/508 (0.6)	55/10973 (0.5)	99/10208 (1.0)	9/663 (1.4)	3/216 (1.4)	169/22568 (0.7)	< 0.001
Perpetrated any IPVA	Yes	79/509 (15.5)	1939/11112 (17.4)	1610/10305 (15.6)	117/667 (17.5)	51/221 (23.1)	3796/22814 (16.6)	< 0.001
IPVA	No experience or perpetration of IPVA	393/509 (77.2)	8429/11074 (76.1)	7824/10286 (76.1)	501/666 (75.2)	153/221 (69.2)	17,300/22756 (76.0)	<.001
	Experienced IPVA only	37/509 (7.3)	724/11074 (6.5)	856/10286 (8.3)	48/666 (7.2)	17/221 (7.7)	1682/22756 (7.4)	
	Perpetrated IPVA only	28/509 (5.5)	795/11074 (7.2)	506/10286 (4.9)	36/666 (5.4)	19/221 (8.6)	1384/22756 (6.1)	
	Both experience and perpetrated IPVA	51/509 (10.0)	1126/11074 (10.2)	1100/10286 (10.7)	81/666 (12.2)	32/221 (14.5)	2390/22756 (10.5)	

There were significant differences by relationship grouping during the past month of COVID-19 restrictions for experiencing financial abuse, experiencing controlling behaviors, perpetrating emotional abuse, experiencing and/or perpetrating technology-facilitated abuse, any physical abuse, sexual abuse and reporting both experiencing and perpetrating IPVA. Non-binary respondents or respondents with non-binary partners reported experiencing most financial abuse, controlling behavior, technology-facilitated abuse and physical abuse. Men partnered with men reported the highest prevalence of experiencing sexual abuse. Non-binary respondents or respondents with non-binary partners reported the highest perpetration of emotional abuse, technology-facilitated abuse, physical abuse and sexual abuse. Women partnered with women also reported a high prevalence of physical abuse perpetration and men partnered with men also reported a high prevalence of sexual abuse perpetration. The highest proportion reporting both experiencing and perpetrating IPVA were those who were non-binary or who had non-binary partners (Table 3).

The experience and use of any abusive behavior during the past 30 days of COVID-19 restrictions are presented by relationship grouping for the 345 respondents who are non-binary or had a non-binary partner in Table 4. Men with non-binary partners (34.4%) and non-binary respondents partnered with men (24.3%) reported the highest proportion of experiencing IPVA during restrictions. A greater proportion of non-binary respondents who were partnered with men (31.9%) and women with non-binary partners (30.8%) reported perpetrating IPVA during the same time period (Table 4).

**Table 4:** Experience and use of intimate partner violence and abuse (IPVA) among respondents who are non-binary or have a non-binary partners during the past 30 days of COVID-19 restrictions (n = 345)

	Women partnered with non-binary partner (*n* = 47)	Men partnered with non-binary partner (*n* = 51)	Non-binary partnered with women (*n* = 97)	Non-binary partnered with men (*n* = 100)	Non-binary with non-binary partner (*n* = 50)
Experienced any IPVA	5/26 (19.2)	11/32 (34.4)	11/63 (17.5)	18/74 (24.3)	5/32 (15.6)
Perpetrated any IPVA	8/26 (30.8)	5/31 (16.1)	9/62 (14.5)	23/72 (31.9)	6/30 (20.0)

Table 5 describes IPVA experience and perpetration during the past 30 days of COVID-19 restrictions for the nine unique gender pairings/groups. Recent experience of IPVA was highest among transgender/non-binary respondents partnered with cis women (24.7%) or cis men (20.6%); and cis men partnered with cis men (20.3%) (Table 5). Recent IPVA perpetration was highest among transgender/non-binary respondents partnered with cis men (24.8%) and cis women with transgender/non-binary partners (23.0%). Both experiencing and perpetrating IPVA was highest among transgender/non-binary respondents partnered with cis men (13.9%). As the number of respondents in groups with transgender /non-binary respondents was low (75–180), differences were not tested for significance.

**Table 5:** Experience and use of intimate partner violence and abuse (IPVA) by gender of respondent and their partner gender of respondent and their partner during the past 30 days of COVID-19 restrictions (n = 35,419)

Variable, n(%)		Cis man- Cis woman (n = 16,615)	Cis man - Cis man (*n* = 956)	Cis woman - Cis man (n = 16,512)	Cis woman - Cis woman (*n* = 671)	Cis man - transgender/non-binary (*n* = 180)	Cis woman - transgender/non-binary (*n* = 125)	Transgender/non-binary - cis man (*n* = 147)	Transgender/non-binary - cis woman (*n* = 138)	Transgender/non-binary - transgender/non-binary (*n* = 75)	Sample (n = 35,419)
Experienced any IPVA	Yes	1962/10376 (18.9)	128/632 (20.3)	1869/11255 (16.6)	81/469 (17.3)	18/96 (18.8)	11/74 (14.9)	22/107 (20.6)	22/89 (24.7)	8/50 (16.0)	4121/23148 (17.8)
Perpetrated any IPVA	Yes	1588/10153 (15.6)	113/620 (18.2)	1907/10927 (17.5)	69/459 (15.0)	13/93 (14.0)	17/74 (23.0)	25/101 (24.8)	14/86 (16.3)	9/47 (19.1)	3755/22560 (16.6)
IPVA	No experience or perpetration of IPVA	7711/10134 (76.1)	460/619 (74.3)	8288/10890 (76.1)	355/459 (77.3)	70/93 (75.3)	54/74 (73.0)	69/101 (68.3)	61/86 (70.9)	37/47 (78.7)	17,105/22503 (76.1)
	Experienced IPVA	839/10134 (8.3)	46/619 (7.4)	713/10890 (6.5)	35/459 (7.6)	10/93 (10.8)	3/74 (4.1)	7/101 (6.9)	11/86 (12.8)	1/47 (2.1)	1665/22503 (7.4)
	Perpetrated IPVA	499/10134 (4.9)	34/619 (5.5)	782/10890 (7.2)	24/459 (5.2)	5/93 (5.4)	9/74 (12.2)	11/101 (10.9)	4/86 (4.7)	3/47 (6.4)	1371/22503 (6.1)
	Both experience and perpetrated IPVA	1085/10134 (10.7)	79/619 (12.8)	1107/10890 (10.2)	45/459 (9.8)	8/93 (8.6)	8/74 (10.8)	14/101 (13.9)	10/86 (11.6)	6/47 (12.8)	2362/22503 (10.5)

Of those who had experienced or perpetrated IPVA during restrictions, 38.2% (1413/3700) reported experience of IPVA and 37.6% (1297/3451) reported perpetration of IPVA had increased since before the restrictions. Although there were no statistically significant differences by relationship grouping, the increase in experiencing or perpetrating IPVA was highest among non-binary respondents or respondents with non-binary partners (44.4 and 54.4% respectively; Additional file 1 Supplementary Table 2).

### Predictors of IPVA

Variables associated with an increase in experiencing IPVA and with an increase in IPVA perpetration during restrictions in bivariate tests (Table 6) were entered into multivariable models (Table 7). Higher psychological distress, not coping well/coping somewhat well with pandemic-related changes (compared to coping very well), some/a lot of relationship tension during COVID-19 restrictions (compared to no tension), and reporting increases and decreases and increases in alcohol consumption during COVID-19 restrictions (compared to no change in alcohol consumption) were significantly associated (*p* < 0.05) with an increase in experiencing IPVA during COVID-19 restrictions in the multivariate model. Respondents who were Black or African American were less likely than respondents who were White to report an increase in experiencing IPVA (Table 7).

**Table 6:** Univariate logistic regression model to assess predictors of an increase in experiencing or perpetrating intimate partner violence and abuse (IPVA)

Predictors	Any increase in experiencing IPVA		Any increase in perpetrating IPVA	
	OR (95% CI)	*P* value	OR (95% CI)	*P* value
Age (*n* = 3707*/3455^†^)	1.00 (0.99, 1.00)	0.364	0.99 (0.99, 1.00)	0.003
Ethnicity (*n* = 3283/3035)				
Black/ African American	0.27 (0.08, 0.93)	0.059	0.96 (0.42, 2.19)	0.282
Asian	0.33 (0.09, 1.13)		0.86 (0.29, 2.51)	
Hispanic/Latino	0.93 (0.51, 1.69)		0.78 (0.43, 1.42)	
Mixed	1.17 (0.82, 1.65)		1.50 (1.06, 2.12)	
Other	0.88 (0.47, 1.65)		0.92 (0.48, 1.77)	
White (ref)				
K6 total score (*n* = 3600*/3370^†^)	1.08 (1.07, 1.09)	< 0.001	1.08 (1.07, 1.10)	< 0.001
Relationship groups (*n* = 3700*/3451^†^)				
Women partnered w/women	1.09 (0.68, 1.73)	0.912	1.31 (0.82, 2.10)	0.141
Women partnered w/men	0.98 (0.85, 1.12)		1.04 (0.90, 1.21)	
Men partnered w/men	1.01 (0.69, 1.47)		0.97 (0.65, 1.44)	
Non-binary	1.29 (0.71, 2.34)		2.05 (1.14, 3.70)	
Men partnered w/women (ref)				
Displaced due to the COVID-19 (*n* = 3421*/3170^†^)				
No, I relocated voluntarily	1.11 (0.80, 1.53)	0.591	1.38 (0.98, 1.92)	0.062
No, I am stranded	1.62 (0.75, 3.51)		2.26 (0.95, 5.38)	
No, situation unrelated to	0.94 (0.57, 1.55)		1.24 (0.77, 2.00)	
COVID-19				
Yes (ref)				
Live with children (*n* = 3412*/3190^†^)				
Yes	1.27 (1.10, 1.46)	0.001	1.12 (0.97, 1.30)	0.123
No (ref)				
Area of residence (*n* = 3420*/3172^†^)				
Rural area	0.94 (0.69, 1.27)-	0.465	0.63 (0.44, 0.91)	0.003
Regional area	0.90 (0.77, 1.07)		0.80 (0.67, 0.95)	
Urban area (ref)				
Change in expenditure (*n* = 3674*/3428^†^)				
I/we have more now	0.84 (0.70, 1.01)	0.002	1.14 (0.95, 1.37)	< 0.001
I/we have less now	1.18 (1.01, 1.36)		1.51 (1.29, 1.77)	
No change (ref)			–	
Financial strain (*n* = 3685*/3443^†^)				
Very difficult	1.60 (1.08, 2.39)	< 0.001	1.20 (0.75, 1.93)	
Difficult	1.43 (1.08, 1.89)		1.18 (0.87, 1.59)	
Somewhat difficult	1.43 (1.18, 1.72)		1.21 (0.99, 1.48)	
Not very difficult (ref)			–	0.196
Coping with changes related to the pandemic (*n* = 3697*/3446^†^)				
Not coping well	4.22 (2.95, 6.05)	< 0.001	4.06 (2.78, 5.93)	< 0.001
Coping somewhat well	2.11 (1.83, 2.43)		1.98 (1.71, 2.30)	
Coping really well (ref)			–	
Relationship tension (*n* = 3704*/3451^†^)				
A lot of tension	8.74 (6.96, 10.98)	< 0.001	5.91 (4.72, 7.40)	< 0.001
Some tension	2.17 (1.77, 2.67)		2.25 (1.85, 2.74)	
No tension (ref)				
Change in alcohol consumption (*n* = 3542*/3326^†^)				
Only decreased	1.16 (0.95, 1.42)	< 0.001	1.23 (0.99, 1.51)	< 0.001
Only increased	1.56 (1.31, 1.86)		1.61 (1.33, 1.94)	
Increased and decreased	1.48 (1.10, 1.98)		1.56 (1.15, 2.10)	
No change (ref)				

*Number of observations in IPVA victimization model

^†^Number of observations in IPVA perpetration model

**Table 7:** Final multivariable logistic regression model to assess predictors of an increase in experiencing or perpetrating intimate partner violence and abuse (IPVA) during COVID-19 restrictions

Predictors	Any increase in experiencing IPVA (*n* = 2782)*		Any increase in perpetrating IPVA (*n* = 2725)^†^	
	OR (95% CI)	*P* value	OR (95% CI)	*P* value
Age	–	–	0.99 (0.98–1.00)	.033
Ethnicity				
Black/African American	0.19 (0.04, 0.89)	.035		
Asian	0.46 (0.09, 2.27)	.341		
Hispanic/Latino	1.03 (0.50, 2.12)	.935		
Mixed	0.99 (0.65, 1.50)	.944		
Other	0.75 (0.34, 1.65)	.481		
White (ref)				
Kessler 6 Distress Scale total score	1.03 (1.01, 1.05)	.002	1.03 (1.01, 1.06)	.003
Relationship groups				
Women partnered w/women			0.77 (0.41, 1.43)	0.405
Women partnered w/men			0.86 (0.72, 1.03)	0.106
Men partnered w/men			0.88 (0.53, 1.44)	0.602
Non-binary			1.60 (0.77, 3.30)	0.207
Men partnered w/women (ref)				
Currently living in your regular place of residence				
No, I relocated voluntarily due to the COVID-19			1.42 (0.97, 2.08)	.074
No, I am stranded by the COVID-19 restrictions			1.94 (0.69, 5.51)	.211
No, but this situation is unrelated to COVID-19			1.23 (0.70, 2.18)	.467
Yes (ref)				
Live with children				
Yes	1.05 (0.89, 1.25)	.560	1.12 (0.93, 1.34)	.243
No (ref)				
Area of residence				
Rural area			0.64 (0.42, 0.98)-	.038-
Regional area			0.84 (0.69, 1.03)	.092
Urban area (ref)				
Amount of money left after expenses changed compared with before COVID-19				
I/we have more now	0.89 (0.71, 1.12)	.336	1.21 (0.98, 1.51)	.083
I/we have less now	0.95 (0.78, 1.16)	.630	1.54 (1.26, 1.88)	<.001
No change (ref)				
Financial strain				
Very difficult	1.08 (0.64, 1.85)	.769	0.54 (0.29, 1.01)	.055
Difficult	1.07 (0.71, 1.62)	.737	0.59 (0.40, 0.87)	.008
Somewhat difficult	1.24 (0.97, 1.60)	.091	0.88 (0.68, 1.14)	.339
Not very difficult (ref)				
Coping with changes related to the pandemic				
Not coping well	1.98 (1.20, 3.26)	.008	2.11 (1.28, 3.48)	.003
Coping somewhat well	1.70 (1.40, 2.06)	<.001	1.44 (1.19, 1.74)	<.001
Coping really well (ref)				
Relationship tension				
A lot of tension	6.78 (5.15, 8.93)	<.001	5.72 (4.36, 7.52)	<.001
Some tension	1.96 (1.53, 2.49)	<.001	2.29 (1.82, 2.89)	<.001
No tension (ref)				
Change in alcohol consumption				
Only decreased	1.25 (0.98, 1.60)	.076	1.37 (1.06, 1.77)	.015
Only increased	1.41 (1.14, 1.75)	.002	1.52 (1.22, 1.91)	<.001
Increased and decreased	1.48 (1.04, 2.11)	.030	1.42 (1.00, 2.02)	.050
No change (ref)				

Notes: OR: odds ratio; CI: confidence intervals

*Ethnicity, K-6 total score, lives with children, area of residence, change in money left after expenses compared with before the COVID-19 restrictions, difficulty in paying for the very basics in the past month, difficulty coping with changes related to the pandemic, relationship tension in past 30 days and change in alcohol consumption were entered into the model to assess predictors of an increase in IPVA victimization during COVID-19 restrictions

^†^Age, relationship group, K-6 total score, lives with children, area of residence, currently living in regular place of residence, change in money left after expenses compared with before the COVID-19 restrictions, difficulty in paying for the very basics in the past month, difficulty coping with changes related to the pandemic, relationship tension in past 30 days and change in alcohol consumption entered into the model assess predictors of an increase in IPVA perpetration during COVID-19 restrictions

Younger age, higher psychological distress, having less money left after expenses (compared to no change in money available), not coping well or coping with some things but not others with pandemic-related changes (compared to coping very well), some/a lot of relationship tension during COVID-19 restrictions (compared to no tension), and reporting any changes (increases; decreases and increases; and decreases and increases) in alcohol consumption during COVID-19 restrictions (compared to no change) were significantly associated (p < 0.05) with an increase in IPVA perpetration during COVID-19 restrictions in the multivariate model. Those who lived in a remote or rural area (compared to urban) or who found it difficult to pay for the very basics in the past month (compared to those who did not) were less likely to report an increase in IPVA perpetration (Table 7).

## Discussion

This study confirmed that IPVA can occur in all intimate relationships, regardless of the perpetrator’s or survivor’s gender. Almost 20% of respondents had experienced or perpetrated IPVA during COVID-19 restrictions. We found differences in the rates of experiencing and perpetrating IPVA during a month of COVID-19 restrictions by relationship groupings. Respondents who were transgender or MSM reported the highest experience of IPVA during the pandemic. Pre-pandemic reviews concluded that transgender individuals were at 2 to 3 times higher risk of physical and sexual IPV compared with cisgender individuals [21]. The comparison of our findings with other studies conducted during the pandemic was not possible for all relationship groupings, due to the lack of studies among SGM. In the US, 23% of men and 15% of women reported experiencing IPVA during early stages of the pandemic [3]. However, the gender of their partners was not recorded. Similar to our findings (17%), some studies conducted during restrictions found that 13–18% of women partnered with men had experienced IPVA [2, 4, 10] but other studies reported a far higher prevalence [5–7]. Studies of MSM conducted during the pandemic reported a similar prevalence of experiencing IPVA (13–15% compared to 18%) [15, 16]. One study found that in male couples, having additional sexual partners outside the relationship during restrictions increased the risk of experiencing IPVA among those with non-monogamous sexual agreements [15]. The authors suggest that this may result from conflict about the risks and severity of COVID-19 impacting on relationship quality. The use of alcohol among MSM has been associated with IPVA perpetration, including sexual IPVA [37]. Studies conducted during COVID-19 have reported increased alcohol and drug use and mental health problems among LGBTQI+ people [15, 16, 38–41]. Therefore, it is likely that the risk of experiencing or perpetrating IPVA during the pandemic would be higher among people in LGBTQI+ relationships.

Similar to one US study [3], we found slightly higher rates of experiencing IPVA among men partnered with women than women partnered with men. Survey methodologies have been criticised for showing ‘gender symmetry’ in experiencing IPVA in heterosexual relationships. Surveys reveal nothing about the perpetrator’s motive and fail to capture context and/or patterns of IPVA [42, 43]. Differences exist in the amount, severity and impact of IPVA, with women more likely to experience fear, repeated abuse, injury, sexual abuse and be killed by a male partner [44–48].

In our study, 23.1% of respondents who were non-binary or had a non-binary partner, 17.5% of men partnered with men, 17.4% of women partnered with men, 15.6% of men partnered with women, 15.5% of women partnered with women had perpetrated IPVA during COVID-19 restrictions. Our finding that 17.5% of men partnered with men had perpetrated IPVA is comparable with a US study that found 15% of cis men in a relationship with a man had perpetrated IPVA during restrictions [15].

Non-binary respondents or respondents with non-binary partners were most likely to perpetrate or experience IPVA. The overreliance on binary gender measurement in research means that relatively little is known about the health and other risk factors and behaviors of non-binary people [34–36, 49–51]. The higher prevalence of IPVA among SGM respondents compared to heterosexual respondents in our sample mirrors pre-pandemic studies findings [21, 52–55]. Non-binary people experience more stressors than transgender men and women and cis people, including greater rates of trauma and interpersonal violence that may contribute to the higher rates of anxiety, depression and psychological distress reported in our survey and in other studies [56]. Internalised (stigma, concealment) and externalised (victimization, discrimination, harassment) minority stressors are associated with IPVA in Lesbian, gay, bisexual, transgender and/or other sexual and gender minority (LGBTQI+) relationships [56–62] and may further explain the excess burden of psychological distress we identified among our non-binary participants. Social and cultural connectedness, specifically support from LGBTQI+ communities [63, 64], protect against the negative health sequelae of gender minority stress [56, 57, 62–65]. One study found that COVID-related stress was higher among SGM than cis heterosexual people [17], and another found that bisexual people or those with other identities experienced higher levels of stress than gay men and lesbians [66]. Stay-at-home orders and social distancing may have disproportionately affected SGM people who have a particular need for connectedness [65], supporting the finding that 34% of the non-binary group reported severe psychological distress.

We found higher proportions of women and non-binary individuals in our study met criteria for psychological distress during the pandemic. Psychological distress was also associated with increases in both experiencing and perpetrating IPVA. There has been a global increase in depression and anxiety reported during the COVID-19 pandemic, compared to pre-pandemic associated with infection, lockdowns, stay-at-home orders, decreased mobility, school and business closures, and decreased social interactions, among other factors [67]. These increases were higher in women than men [67, 68], potentially due to women experiencing greater social and economic consequences during the pandemic, including additional carer and household responsibilities, less secure employment and experience of IPVA. Studies of GSM report higher depressive symptoms than cis people pre and during the pandemic [69–71]. Mental health impacts of the pandemic have been experienced more by those with existing mental health issues [72]. Reduced ability of transgender and nonbinary people to live according to their gender, including access to gender-affirming resources, medication and transition-related care has been reported during COVID-19 [73] and associated with increased gender dysphoria and depression [73].

40 % of our sample reported an increase on all three alcohol measures during the pandemic, the highest proportion was among men partnered with men (42.9%). Regardless of gender or sexual orientation, studies have reported increases in depression during COVID-19 [39, 72, 74]. Moreover, increased stress, depression and reduced psychological wellbeing were associated with increased drinking during the pandemic [75–77] to cope with feelings of boredom, stress, loneliness and depression [74, 78]. People with existing mental health conditions (including in the GDS) reported greater increases in drinking during the pandemic [74, 79]. Among men partnered with men in our sample, increased drinking potentially contributed to the higher prevalence of experiencing sexual abuse [74, 80, 81]. One study reported that experiencing IPVA during restrictions was positively associated with the number of casual sexual partners that men in a relationship with men met during the pandemic [15]. Casual sex and sexualised drug use have remained common among MSM during the pandemic [16, 82–84] and may have contributed to sexual abuse.

The majority of IPVA survivors or perpetrators during COVID-19 restrictions reported that this had stayed the same or decreased compared to February 2020. However, 38.1% of survivors and 37.6% of perpetrators reported that their experience or perpetration of IPVA respectively had increased compared to pre-pandemic. We found that higher psychological distress, not coping well/coping somewhat well with pandemic-related changes, some/a lot of relationship tension during COVID-19 restrictions, changes in alcohol consumption, younger age (perpetration only) and having less money left after expenses compared with before COVID-19 (perpetration only) were associated with an increase in experiencing or perpetrating IPVA during restrictions. Stay-at-home orders, remote working and social distancing have made it easier for perpetrators to abuse and monitor their partners, with many survivors isolated and unable to access (in) formal support [2, 85–87]. While COVID-19 does not cause IPVA [88], the pandemic has resulted in economic insecurity or financial difficulties, increased stress, caring responsibilities, mental health problems and alcohol use for many [39, 74, 89, 90]. These documented risk factors for IPVA [91–94] have been associated with increases in IPVA during COVID-19 [3–10, 17, 95] and following natural disasters [96–98].

### Strengths and limitations

With almost 36,000 respondents, this is the largest study conducted on IPVA during the pandemic (other studies sample sizes range from 560 [4]–15,000 [2]), and the only one comparing IPVA by different relationship groupings. Due to the small sample size for some relationship groupings, we were not able to analyze all relationship dyads. The large sample of cis men and cis women means that ‘small’ differences between these groups – compared to transgender or binary groups - are likely to be statistically significant. Transgender and non-binary people contribute < 1% of population-based studies [99] and many studies of IPVA in transgender people report smaller or comparable sample sizes [21] to our survey. Despite the small number in some relationship groupings, given the lack of comparative studies, being able to make the comparison with other groups is of significant value and therefore an acceptable limitation. The inclusion of non-binary respondents is a strength. Had binary transgender people been classified alongside non-binary respondents, the finding that the experience and perpetration of IPVA increased in the non-binary group would have been obscured. As the GDS collected data from a non-probability sample recruited through media outlines, social networks, and word-of-mouth; this may have resulted in a greater proportion of LGBTQI+ respondents participating than probability sampling would have included. Generally, the GDS recruits a higher proportion of LGBTQI+ (around 15%) people than a probability or representative sample would. The GDS is designed to answer comparison questions that are not dependent on probability samples. When the aims of research are to examine the relationships between variables or in-depth profiling of sub groups, it has been argued that probability-based sampling may be unnecessary or even better avoided [100, 101]. The GDS excludes people who do not use licit and/or illicit substances, including alcohol, and who have access to and use the internet, resulting in a younger sample with high levels of health literacy. Moreover, response bias is more likely as people respond to surveys if they perceive the subject to be of interest to them, and therefore their characteristics may differ from those who chose not to participate.

However, at no cost to governments, the GDS facilitates the inclusion of more hidden and hard-to-reach populations, that general. More cost-prohibitive, household surveys do not.

[27]. The survey required people to respond based on their experiences in the past month (May or June 2020) compared with the month of February 2020, which may have resulted in recall bias.

Optional questions on relationships were placed at the end of the survey for respondents currently in a relationship. One limitation was the moderate quantity of missing data. A complete case approach was taken under the assumption that respondents with missing data were a random sample of the full dataset, which may have led to bias in estimates. Furthermore, to avoid inflating any analytical problems due to small-cell sizes (noted earlier), data were not clustered by country.

An adapted CASR-SF measured IPVA. The CASR-SF was validated in women victims [31], therefore further research is required to examine how it performs among SGM.

### Implications for public health

The need to provide IPVA and mental health support to meet the needs of all genders and sexualities during the pandemic has been demonstrated [67]. Most IPVA victim support services have been designed for heterosexual, cis women, making them ‘invisible’, less accessible or inclusive for cis heterosexual men and LGBTQI+ people [102–105]. Staff often make assumptions about heterosexuality, lack understanding about IPVA in LGBTQI+ relationships and there is a lack of appropriate referral pathways [103, 105]. Male and LGBTQI+ victims report specific barriers in help-seeking including shame and denial (men), internalised stigma (LGBTQI+ people), failure to recognise their experience as IPVA and negative experiences or perceptions of support [103, 104]. In addition, there are a lack of perpetrator programmes for women or people who are LGBTQI+ [106]. IPVA support services and perpetrator programmes must be tailored to support all perpetrators and survivors during the pandemic and beyond, regardless of their sexual or gender identity. Stay-at-home orders and social distancing have highlighted the need to provide support for survivors and perpetrators digitally [107]. Providing such support digitally can improve access, especially for those who live in remote communities or in areas with few services, and offer more flexibility for people [107–109], during and post-pandemic. Digital literacy and poverty need to be considered to enhance engagement.

Given the increased psychological distress exhibited by transgender and non-binary people, improved access to gender-affirming resources is required [73].

### Implications for future research

We found differences in the use and experience of different types of IPVA during COVID-19 among different relationship groupings. There remains a need to qualitatively explore the contextual factors and relationship dynamics in non-heterosexual relationships to better understand IPVA and how best to address it.

## Conclusions

IPVA occurs in all intimate relationships. Victim support and perpetrator interventions should available to all, regardless of the survivor’s or perpetrator’s gender. Cisgender women.

## Supplementary Information

**Additional file 1.** Additional file.

## Data Availability

Data and materials are available from the authors on request.

## Abbreviations

cis: Cisgender
CASR-SF: Composite Abuse Scale Revised–Short Form
GDS: Global Drug Survey
IPVA: Intimate partner violence and abuse
IQRs: interquartile ranges
K-6: Kessler 6 Psychological Distress Scale
LGBTQI+: Lesbian, gay, bisexual, transgender and/or other sexual and gender minority
MSM: Men who have sex with men
sd: standard deviation
SGM: Sexual or gender minority
WSW: Women who have sex with women.
