# Supplementary material for: Experience and perpetration of intimate partner violence and abuse by gender of respondent and their current partner before and during COVID-19 restrictions in 2020: a cross-sectional study in 13 countries
Source: BMC Public Health. 2023 Feb 13;23:316. doi: 10.1186/s12889-022-14635-2 (PMC9924203; doi:10.1186/s12889-022-14635-2)
Supplement: Supplementary file 1 — Additional file 1. Additional file. [file 12889_2022_14635_MOESM1_ESM.docx]

**Supplementary Table 1. Survey questions and response options**

| **Survey questions** | **Response options** |
| --- | --- |
| **Sexuality and gender** |  |
| What is your gender? | - Male - Female - Non-binary - Different identity |
| What gender were you assigned at birth? | - Male - Female |
| Do you consider yourself to be: | - Straight or heterosexual - Lesbian, gay or homosexual - Bisexual Queer - Different orientation - Don't know/prefer not to say |
| What gender is your partner/ boyfriend/ girlfriend? | - Male - Female - Non-binary - Different identity |
| What gender was your partner assigned at birth? | - Male - Female |
| **Ethnicity** |  |
| What is your ethnicity? | - White - Black / African American - Asian - Hispanic/Latino - Aboriginal/Maori - Native American - Mixed - Other - Please specify |
| **Area of residence** |  |
| Are you currently living in your regular place of residence? | - Yes - No, I relocated voluntarily due to the COVID-19 pandemic/ restrictions - No, I am stranded by the COVID-19 movement restrictions - No, but this situation is unrelated to COVID-19 |
| Would you say you are living in a... | - City/urban area - Regional area - Remote/rural area |
| **Employment** |  |
| Are you currently in paid employment? | - Yes, full-time - Yes, part-time/ casual - No |
| **Financial situation** |  |
| Has the amount of money you have left after expenses changed compared with before the COVID-19 restrictions? *If your household shares finances, consider the financial situation of your household*. | - No change - I/We have more now - I/We have less now |
| In the past month, how difficult has it been for you to pay for the very basics like food, housing, medical care, and heating? | - Very difficult - Difficult - Somewhat difficult - Not very difficult |
| **Relationships** |  |
| Are you currently in an adult intimate relationship? *By adult intimate relationship we mean a husband/wife, partner or boyfriend/girlfriend for longer than one month.* | - Yes, with 1 partner - Yes, with 2 or more partners - No |
| How would you describe your relationship with your partner/boyfriend/girlfriend in February—before the COVID-19 restrictions? | - No tension - Some tension - A lot of tension - Not applicable – I did not have this partner/boyfriend/girlfriend in February |
| How would you describe your relationship with your partner/boyfriend/girlfriend in the last 30 days? | - No tension - Some tension - A lot of tension |
| **Experience of abusive behaviour** |  |
| In the past 30 days, my partner/boyfriend/girlfriend kept me from having access to a job, money or financial resources | - Not in the past 30 days - Once - A few times (2-3 times) - Weekly / almost weekly - Daily / almost daily |
| If yes, compared to February—before the COVID-19 restrictions, has this behaviour changed? | - Increased - Stayed the same - Decreased - Don’t know/Unsure - Not applicable – I did not have this partner/boyfriend/girlfriend in February |
| In the past 30 days, my partner/boyfriend/girlfriend told me I was crazy, stupid or not good enoug | - Not in the past 30 days - Once - A few times (2-3 times) - Weekly / almost weekly - Daily / almost daily |
| If yes, compared to February—before the COVID-19 restrictions, has this behaviour changed? | - Increased - Stayed the same - Decreased - Don’t know/Unsure - Not applicable – I did not have this partner/boyfriend/girlfriend in February |
| In the past 30 days, my partner/boyfriend/girlfriend kept me from seeing or talking to my family or friends | - Not in the past 30 days - Once - A few times (2-3 times) - Weekly / almost weekly - Daily / almost daily |
| If yes, compared to February—before the COVID-19 restrictions, has this behaviour changed? | - Increased - Stayed the same - Decreased - Don’t know/Unsure - Not applicable – I did not have this partner/boyfriend/girlfriend in February |
| In the past 30 days, my partner/boyfriend/girlfriend checked up on me by checking my phone, text, email or social media without my consent | - Not in the past 30 days - Once - A few times (2-3 times) - Weekly / almost weekly - Daily / almost daily |
| If yes, compared to February—before the COVID-19 restrictions, has this behaviour changed? | - Increased - Stayed the same - Decreased - Don’t know/Unsure - Not applicable – I did not have this partner/boyfriend/girlfriend in February |
| In the past 30 days, my partner/boyfriend/girlfriend shook, pushed, grabbed or threw me | - Not in the past 30 days - Once - A few times (2-3 times) - Weekly / almost weekly - Daily / almost daily |
| If yes, compared to February—before the COVID-19 restrictions, has this behaviour changed? | - Increased - Stayed the same - Decreased - Don’t know/Unsure - Not applicable – I did not have this partner/boyfriend/girlfriend in February |
| In the past 30 days, my partner/boyfriend/girlfriend hit me with a fist or object, kicked or bit me | - Not in the past 30 days - Once - A few times (2-3 times) - Weekly / almost weekly - Daily / almost daily |
| If yes, compared to February—before the COVID-19 restrictions, has this behaviour changed? | - Increased - Stayed the same - Decreased - Don’t know/Unsure - Not applicable – I did not have this partner/boyfriend/girlfriend in February |
| In the past 30 days, my partner/boyfriend/girlfriend threatened to harm or kill me or someone close to me | - Not in the past 30 days - Once - A few times (2-3 times) - Weekly / almost weekly - Daily / almost daily |
| If yes, compared to February—before the COVID-19 restrictions, has this behaviour changed? | - Increased - Stayed the same - Decreased - Don’t know/Unsure - Not applicable – I did not have this partner/boyfriend/girlfriend in February |
| In the past 30 days, my partner/boyfriend/girlfriend forced or tried to force me to have sex | - Not in the past 30 days - Once - A few times (2-3 times) - Weekly / almost weekly - Daily / almost daily |
| If yes, compared to February—before the COVID-19 restrictions, has this behaviour changed? | - Increased - Stayed the same - Decreased - Don’t know/Unsure - Not applicable – I did not have this partner/boyfriend/girlfriend in February |
| **Use of abusive behaviours** |  |
| In the past 30 days, I kept my partner/ boyfriend/ girlfriend from having access to a job, money or financial resources | - Not in the past 30 days - Once - A few times (2-3 times) - Weekly / almost weekly - Daily / almost daily |
| If yes, compared to February—before the COVID-19 restrictions, has this behaviour changed? | - Increased - Stayed the same - Decreased - Don’t know/Unsure - Not applicable – I did not have this partner/boyfriend/girlfriend in February |
| In the past 30 days, I told my partner/ boyfriend/ girlfriend they were crazy, stupid or not good enough | - Not in the past 30 days - Once - A few times (2-3 times) - Weekly / almost weekly - Daily / almost daily |
| If yes, compared to February—before the COVID-19 restrictions, has this behaviour changed? | - Increased - Stayed the same - Decreased - Don’t know/Unsure - Not applicable – I did not have this partner/boyfriend/girlfriend in February |
| In the past 30 days, I kept my partner/ boyfriend/ girlfriend from seeing or talking to their family or friends | - Not in the past 30 days - Once - A few times (2-3 times) - Weekly / almost weekly - Daily / almost daily |
| If yes, compared to February—before the COVID-19 restrictions, has this behaviour changed? | - Increased - Stayed the same - Decreased - Don’t know/Unsure - Not applicable – I did not have this partner/boyfriend/girlfriend in February |
| In the past 30 days, I kept my partner/ boyfriend/ girlfriend from seeing or talking to their family or friends | - Not in the past 30 days - Once - A few times (2-3 times) - Weekly / almost weekly - Daily / almost daily |
| If yes, compared to February—before the COVID-19 restrictions, has this behaviour changed? | - Increased - Stayed the same - Decreased - Don’t know/Unsure - Not applicable – I did not have this partner/boyfriend/girlfriend in February |
| In the past 30 days, I shook, pushed, grabbed or threw my partner/ boyfriend/ girlfriend | - Not in the past 30 days - Once - A few times (2-3 times) - Weekly / almost weekly - Daily / almost daily |
| If yes, compared to February—before the COVID-19 restrictions, has this behaviour changed? | - Increased - Stayed the same - Decreased - Don’t know/Unsure - Not applicable – I did not have this partner/boyfriend/girlfriend in February |
| In the past 30 days, I hit my partner/ boyfriend/ girlfriend with a fist or object, kicked or bit them | - Not in the past 30 days - Once - A few times (2-3 times) - Weekly / almost weekly - Daily / almost daily |
| If yes, compared to February—before the COVID-19 restrictions, has this behaviour changed? | - Increased - Stayed the same - Decreased - Don’t know/Unsure - Not applicable – I did not have this partner/boyfriend/girlfriend in February |
| In the past 30 days, I threatened to harm or kill my partner/ boyfriend/ girlfriend or someone close to them | - Not in the past 30 days - Once - A few times (2-3 times) - Weekly / almost weekly - Daily / almost daily |
| If yes, compared to February—before the COVID-19 restrictions, has this behaviour changed? | - Increased - Stayed the same - Decreased - Don’t know/Unsure - Not applicable – I did not have this partner/boyfriend/girlfriend in February |
| In the past 30 days, I forced or tried to force my partner/ boyfriend/ girlfriend to have sex | - Not in the past 30 days - Once - A few times (2-3 times) - Weekly / almost weekly - Daily / almost daily |
| If yes, compared to February—before the COVID-19 restrictions, has this behaviour changed? | - Increased - Stayed the same - Decreased - Don’t know/Unsure - Not applicable – I did not have this partner/boyfriend/girlfriend in February |
| **Alcohol** |  |
| In the last 30 days, on how many days did you drink alcohol? | - 1-30 |
| Compared to February—before the COVID-19 restrictions, has the number of days you drink alcohol in a typical week changed? | - Increased a lot - Increased a little - Stayed the same - Decreased a little - Decreased a lot - Don’t know/Unsure |
| In the last 30 days, how many standard drinks containing alcohol did you have on a typical day? | - 1 or 2 - 3 or 4 - 5 or 6 - 7 to 9 - 10 or more |
| Compared to February—before the COVID-19 restrictions, has the number of standard drinks containing alcohol that you have on a typical day changed? | - Increased a lot - Increased a little - Stayed the same - Decreased a little - Decreased a lot - Don’t know/Unsure |
| In the last 30 days, on how many days did you have five or more drinks on a single occasion? | - 1-30 |
| Compared to February—before the COVID-19 restrictions, has the number of times you had five or more drinks on a single occasion changed? | - Increased a lot - Increased a little - Stayed the same - Decreased a little - Decreased a lot - Don’t know/Unsure |
| **Mental health and coping** |  |
| During the past 30 days, about how often did you feel nervous? | - All of the time - Most of the time - Some of the time - A little of the time - None of the time |
| During the past 30 days, about how often did you feel hopeless? | - All of the time - Most of the time - Some of the time - A little of the time - None of the time |
| During the past 30 days, about how often did you feel restless or fidgety? | - All of the time - Most of the time - Some of the time - A little of the time - None of the time |
| During the past 30 days, about how often did you feel restless or so depressed that nothing could cheer you up? | - All of the time - Most of the time - Some of the time - A little of the time - None of the time |
| During the past 30 days, about how often did you feel that everything was an effort? | - All of the time - Most of the time - Some of the time - A little of the time - None of the time |
| During the past 30 days, about how often did you feel worthless? | - All of the time - Most of the time - Some of the time - A little of the time - None of the time |
| How have you been coping with changes related to the COVID-19 pandemic? | - I’m coping really well - I’m coping with some things but not others - I’m not coping well at all |

**Supplementary Table 2: Changes in experienceExperience and perpetration of intimate partner violence and abuse during COVID-19 restrictions compared to February 2020 (n=35,984)**

| **Variable, n (%)** |  | **Women partnered with women (n=747)** | **Women partnered with men (n=16,805)** | **Men partnered with women (n=16,915)** | **Men partnered with men**  **(n=1,042)** | **Non-binary respondents or respondents with a non-binary partner (n=345)** | **Sample (n=35,854)** | **P value from Fishers Exact test** |
| --- | --- | --- | --- | --- | --- | --- | --- | --- |
| Experience of financial control | Decreased | 1/8(12.5) | 5/73 (6.8) | 3/128 (2.3) | 0/2 (0.0) | 1/5 (20.0) | 10/216 (4.6) | 0.237 |
|  | Stayed the same | 4/8 (50.0) | 30/73 (41.1) | 60/128 (46.9) | 0/2 (0.0) | 2/5 (40.0) | 97/216 (44.7) |  |
|  | Increased | 3/8 (37.5) | 38/73 (52.1) | 65/128 (50.8) | 2/2 (100.0) | 2/5 (40.0) | 110/216 (50.7) |  |
| Experience of emotional abuse | Decreased | 2/48 (4.2) | 87/1232 (7.1) | 54/1112 (4.9) | 4/80 (5.0) | 4/31 (12.9) | 152/2503 (6.1) | *0.264 |
|  | Stayed the same | 29/48 (60.4) | 701/1232 (56.9) | 653/1112 (58.7) | 47/80 (58.8) | 13/31 (41.9) | 1446/2503 (57.7) |  |
|  | Increased | 17/48 (35.4) | 444/1232 (36.0) | 405/1112 (36.4) | 29/80 (36.3) | 14/31 (45.2) | 910/2503 (36.3) |  |
| Experience of coercive control | Decreased | 1/15 (6.7) | 22/239 (9.2) | 23/399 (5.8) | 2/16 (12.5) | 0/13 (0.0) | 48/682 (7.0) | 0.512 |
|  | Stayed the same | 7/15 (46.7) | 108/239 (45.2) | 193/399 (48.4) | 6/16 (37.5) | 4/13 (30.8) | 318/682 (46.6) |  |
|  | Increased | 7/15 (46.7) | 109/239 (45.6) | 183/399 (45.9) | 8/16 (50.0) | 9/13 (69.2) | 316/682 (46.3) |  |
| Experience of threating behaviour | Decreased | 0/1 (0.0) | 3/53 (5.7) | 2/51 (3.9) | 0/7 (0.0) | 0/2 (0.0) | 5/114 (4.4) | 0.278 |
|  | Stayed the same | 1/1 (100.0) | 20/53 (37.7) | 30/51 (58.8) | 4/7(57.1) | 0/2 (0.0) | 55/114 (48.2) |  |
|  | Increased | 0/1 (0.0) | 30/53 (56.6) | 19/51 (37.3) | 3/7 (42.9) | 2/2 (100.0) | 54/114 (47.4) |  |
| Experience of technology facilitated abuse | Decreased | 3/16 (18.8) | 15/230 (6.5) | 21/436 (4.8) | 1/34 (2.9) | 0/10 (0.0) | 40/726 (5.5) | 0.433 |
|  | Stayed the same | 8/16 (50.0) | 163/230 (70.9) | 312/436 (71.6) | 26/34 (76.5) | 7/10 (70.0) | 517/726 (71.1) |  |
|  | Increased | 5/16 (31.3) | 52/230 (22.6) | 103/436 (23.6) | 7/34 (20.6) | 3/10 (30.0) | 170/726 (23.4) |  |
| Experience of physical abuse (shook/pushed/grabbed) | Decreased | 2/17 (11.8) | 11/227 (4.8) | 9/237 (3.8) | 0/14 (0.0) | 1/8 (12.5) | 23/503 (4.6) | 0.068 |
|  | Stayed the same | 11/17 (64.7) | 112/227 (49.3) | 147/237 (62.0) | 9/14 (64.3) | 5/8 (62.5) | 285/503 (56.5) |  |
|  | Increased | 4/17 (23.5) | 104/227 (45.8) | 81/237 (34.2) | 5/14 (35.7) | 2/8 (25.0) | 196/503 (38.9) |  |
| Experience of severe physical abuse (hit with fist or object) | Decreased | 0/6 (0.0) | 6/51 (11.8) | 10/157 (6.4) | 0/12 (0.0) | 0/5 (0.0) | 16/231 (6.9) | 0.262 |
|  | Stayed the same | 2/6 (33.3) | 21/51 (41.2) | 85/157 (54.1) | 7/12 (58.3) | 5/5 (100.0) | 120/231 (51.9) |  |
|  | Increased | 4/6 (66.7) | 24/51 (47.1) | 62/157 (39.5) | 5/12 (41.7) | 0/5 (0.0) | 95/231 (41.1) |  |
| Experience of sexual abuse | Decreased | 0/4 (0.0) | 12/164 (7.3) | 4/100 (4.0) | 0/10 (0.0) | 1/4 (25.0) | 17/282 (6.0) | 0.471 |
|  | Stayed the same | 2/4 (50.0) | 96/164 (58.5) | 67/100 (67.0) | 5/10 (50.0) | 2/4 (50.0) | 172/282 (61.0) |  |
|  | Increased | 2/4 (50.0) | 56/164 (34.1) | 29/100 (29.0) | 5/10 (50.0) | 1/4 (25.0) | 93/282 (33.0) |  |
| Increase in any experience of IPVA (out of those who reported experiencing any IPVA) | No | 46/77 (59.7) | 1038/1669 (62.2) | 1103/1787 (61.7) | 75/122 (61.5) | 25/45 (55.6) | 2287/3700 (61.8) | *0.910 |
|  | Yes | 31/77 (40.3) | 631/1669 (37.8) | 684/1787 (38.3) | 47/122 (38.5) | 20/45 (44.4) | 1413/3700 (38.2) |  |
| Perpetrator of financial control | Decreased | 1/5 (20.0) | 1/49 (2.0) | 1/54 (1.9) | 0 (.) | 0 (.) | 3/108 (2.8) | 0.219 |
|  | Stayed the same | 1/5 (20.0) | 28/49 (57.1) | 29/54 (53.7) | 0 (.) | 0 (.) | 58/108 (53.7) |  |
|  | Increased | 3/5 (60.0) | 20/49 (40.8) | 24/54 (44.4) | 0 (.) | 0 (.) | 47/108 (43.5) |  |
| Perpetrator of emotional abuse | Decreased | 2/42 (4.8) | 61/1057 (5.8) | 44/955 (4.6) | 5/77 (6.5) | 0/31 (0.0) | 112/2162 (5.2) | *0.090 |
|  | Stayed the same | 17/42 (40.5) | 600/1057 (56.8) | 570/955 (59.7) | 44/77 (57.1) | 14/31 (45.2) | 1245/2162 (57.6) |  |
|  | Increased | 23/42 (54.8) | 396/1057 (37.5) | 341/955 (35.7) | 28/77 (36.4) | 17/31 (54.8) | 805/2162 (37.2) |  |
| Perpetrator of coercive control | Decreased | 2/9 (22.2) | 5/162 (3.1) | 5/153 (3.3) | 0/5 (0.0) | 0/7 (0.0) | 12/336 (3.6) | 0.042 |
|  | Stayed the same | 4/9 (44.4) | 63/162 (38.9) | 71/153 (46.4) | 3/5 (60.0) | 0/7 (0.0) | 141/336 (42.0) |  |
|  | Increased | 3/9 (33.3) | 94/162 (58.0) | 77/153 (50.3) | 2/5 (40.0) | 7/7 (100.0) | 183/336 (54.5) |  |
| Perpetrator of threatening behaviour | Decreased | 0/2 (0.0) | 4/24 (16.7) | 3/27 (11.1) | 0/3 (0.0) | 0/1 (0.0) | 7/57 (12.3) | 0.166 |
|  | Stayed the same | 1/2 (50.0) | 13/24 (54.2) | 6/27 (22.2) | 1/3 (33.3) | 0/1 (0.0) | 21/57 (36.8) |  |
|  | Increased | 1/2 (50.0) | 7/24 (29.2) | 18/27 (66.7) | 2/3 (66.7) | 1/1 (100.0) | 29/57 (50.9) |  |
| Perpetrator of technology facilitated abuse | Decreased | 2/26 (7.7) | 41/643 (6.4) | 20/363 (5.5) | 2/36 (5.6) | 1/12 (8.3) | 66/1080 (6.1) | 0.979 |
|  | Stayed the same | 17/26 (65.4) | 430/643 (66.9) | 246/363 (67.8) | 23/36 (63.9) | 7/12 (58.3) | 724/1080 (67.0) |  |
|  | Increased | 7/26 (26.9) | 172/643 (26.7) | 97/363 (26.7) | 11/36 (30.6) | 4/12 (33.3) | 291/1080 (26.9) |  |
| Perpetrator of physical abuse (shook/pushed/grabbed) | Decreased | 3/16 (18.8) | 7/166 (4.2) | 12/188 (6.4) | 0/10 (0.0) | 0/5 (0.0) | 22/385 (5.7) | 0.321 |
|  | Stayed the same | 8/16 (50.0) | 76/166 (45.8) | 94/188 (50.0) | 6/10 (60.0) | 4/5 (80.0) | 188/385 (48.8) |  |
|  | Increased | 5/16 (31.3) | 83/166 (50.0) | 82/188 (43.6) | 4/10 (40.0) | 1/5 (20.0) | 175/385 (45.5) |  |
| Perpetrator of severe physical abuse (hit with fist or object) | Decreased | 1/9 (11.1) | 3/93 (3.2) | 6/61 (9.8) | 0/6 (0.0) | 1/5 (20.0) | 11/174 (6.3) | 0.162 |
|  | Stayed the same | 3/9 (33.3) | 38/93 (40.9) | 31/61 (50.8) | 4/6 (66.7) | 3/5 (60.0) | 79/174 (45.4) |  |
|  | Increased | 5/9 (55.6) | 52/93 (55.9) | 24/61 (39.3) | 2/6 (33.3) | 1/5 (20.0) | 84/174 (48.3) |  |
| Perpetrator of sexual abuse | Decreased | 0/2 (0.0) | 2/44 (4.5) | 3/89 (3.4) | 1/9 (11.1) | 0/2 (0.0) | 6/146 (4.1) | 0.717 |
|  | Stayed the same | 1/2 (50.0) | 28/44 (63.6) | 63/89 (70.8) | 6/9 (66.7) | 2/2 (100.0) | 100/146 (68.5) |  |
|  | Increased | 1/2 (50.0) | 14/44 (31.8) | 23/89 (25.8) | 2/9 (22.2) | 0/2 (0.0) | 40/146 (27.4) |  |
| Increase in any IPVA perpetration (out of those who reported being a perpetrator of any IPVA) | No | 42/74 (56.8) | 1091/1752 (62.3) | 927/1465 (63.3) | 73/114 (64.0) | 21/46 (45.7) | 2154/3451 (62.4) | *0.129 |
|  | Yes | 32/74 (43.2) | 661/1752 (37.7) | 538/1465 (36.7) | 41/114 (36.0) | 25/46 (54.4) | 1297/3451 (37.6) |  |

*Chi2 test
